# Defining Mental Health Conditions Within Primary Care Data: A Validation Study With a Mixed Qualitative and Quantitative Approach

**DOI:** 10.1111/jep.14256

**Published:** 2024-12-11

**Authors:** Juan Carlos Bazo‐Alvarez, Christina Avgerinou, Danielle Nimmons, Joseph F. Hayes, David Osborn, Claudia Cooper, Kate Walters, Irene Petersen

**Affiliations:** ^1^ Research Department of Primary Care and Population Health University College London (UCL) London UK; ^2^ Division of Psychiatry University College London (UCL) London UK; ^3^ Wolfson Institute of Population Health Queen Mary University of London London UK

**Keywords:** codelist, electronic health records, mental health, primary care, Read codes, validation

## Abstract

**Objectives:**

To validate codelists for defining a range of mental health (MH) conditions with primary care data, using a mixed qualitative and quantitative approach and without requiring external data.

**Methods:**

We validated Read codelists, selecting and classifying them in three steps. The qualitative step included an in‐depth revision of the codes by six doctors. Simultaneously, the quantitative step performed on UK primary care data included an exploratory factor analysis to cluster Read codes in MH conditions to obtain an independent classification. The statistical results informed the qualitative conclusions, generating a final selection and classification.

**Results:**

From a preselected list of 2007 Read codes, a total of 1638 were selected by all doctors. Later, they agreed on classifying these codes into 12 categories of MH disorders. From the same preselected list, a total of 1364 were quantitatively selected. Using data from 497,649 persons who used these Read codes at least once, we performed the exploratory factor analysis, retaining five factors (five categories). Both classifications showed good correspondence, while discrepancies informed decisions on reclassification.

**Conclusions:**

We produced a comprehensive set of medical codes lists for 12 MH conditions validated by a combination of clinical consensus panel and quantitative cluster analysis with cross‐validation.

## Summary

In primary care, people's mental health condition is registered using alphanumeric codes (e.g., a number followed by letters). These medical codes are used in health research in the form of codelists, but these lists require a previous formal revision called validation. We aimed to perform this validation without relying on external information (i.e., using primary care data only) since external data can be costly and logistically challenging. We applied a mixed approach, combining qualitative analysis by a panel of six doctors with statistical analysis of a large primary care database (The Health Improvement Network database). From an initial list of 2007 medical codes, 1638 were selected by the doctors and categorised into 12 mental health disorders. Simultaneously, statistical analysis of data from 497,649 individuals identified 5 distinct categories. The final classification, achieved through a combination of both approaches, showed good consistency when contrasted against relevant drug treatment information. This comprehensive set of validated medical codelists for mental health conditions demonstrated a successful blend of clinical consensus and quantitative analysis, maximising the utility of primary care data and avoiding the need for external data collection.

## 1 Introduction

Primary care data have become a valuable resource for mental health (MH) research. These data offer access to individual follow‐up of very large groups of patients over long‐term periods—usually years—and in typical clinical contexts [1, 2, 3]. MH data can be studied together with many health indicators recorded in primary care [4]. Moreover, primary care data can be linked to external data sets (e.g., hospitalisation, genetic or mortality data) to expand the topics of interest studied with observational designs [5, 6, 7].

However, defining MH conditions using primary care data is not straightforward. These data are collected for clinical and administrative purposes, not for research; thus, the recording of MH conditions is not necessarily homogeneous across patients, doctors, general practices, or over time [8, 9, 10]. For example, some doctors may prefer one medical code over others for recording a symptom of depression, while others may not code diagnoses of mental disorders since they can be perceived as stigmatising by patients and doctors. As a result, when conducting epidemiological research using routinely collected primary care data, the identification of MH conditions (e.g., depression) as defined by the standard use of medical diagnosis codes alone can lead to underestimating their actual incidence. Researchers have addressed this problem by using algorithms and codelists for symptoms, treatments and processes [11]. Algorithms detect and integrate data from different sources or coded as different variables to define if the patient has an MH condition. For example, if a medical code of a depressive symptom has been followed by a 6‐month prescription of antidepressant medication, the algorithm defines this as a patient with depression. A list of medical codes usually supplies these algorithms with relevant data to improve the accuracy of case definitions. Given the problem described above, both algorithms and codelists need to be validated in some way [12]. In this study, we focus on the validation of codelists for MH conditions.

The validation of codelists can involve a qualitative or quantitative approach, using either internal or external information to primary care data. In a systematic review, Carreira et al. [13] reported a substantial variability between the codelists used for MH research, with only 30/120 studies performing a validation step. In these studies, clinical review internal validation was the most typical practice; an example of this is when doctors review the cases defined by codelists to ensure they have been correctly classified [14]. However, this type of qualitative validation is not extensive as, in practice, it is only possible to conduct this with random samples of cases. Quantitative analysis can use external information to inform the qualitative review [11]. However, the use of quantitative validation against linked external data is rare since it increases cost, logistics and data management. A mixed qualitative and quantitative approach that only requires information from the same primary care data could reduce barriers and facilitate the validation step for researchers.

We aimed to validate codelists for defining a range of MH conditions with primary care data, using a mixed qualitative and quantitative approach and without the need for external data [11]. We report a set of validated codelists for use by other researchers, and sufficient details to reproduce the validation procedure in future studies.

## 2 Methods

We validated Read codes lists created to define MH conditions in patients registered in the UK primary care system. We used a mixed qualitative and quantitative approach for this validation. The qualitative step included an in‐depth review of the codes by a panel of six doctors (three general practitioners [GPs] and three psychiatrists), whereas the quantitative step included different statistical analyses to inform the qualitative revision. Details about the data and steps are provided in the following sections.

### 2.1 Primary Care Data

Data came from The Health Improvement Network (THIN), one of the largest sources of longitudinal de‐identified electronic health record data in the United Kingdom. The data are collected via the In Practice Systems software called Vision GP. Data from the Vision GP system is sent to Cegedim, the THIN owners, who then supply this data to IQVIA under license. After anonymising data, IQVIA provides and supports access to this data for health research. THIN includes data from 15.6 million patients, of which 3 million are active patients from 711 practices who can be prospectively followed [15]. There are different quality markers for THIN data. The acceptable computer usage (ACU) is defined as ‘the year in which a general practice was continuously entering on average at least two therapy records, one medical record and one additional health data record per patient per year’ [16]. Likewise, there is a quality marker for acceptable mortality reporting (AMR), indicating when GP's mortality records are consistent with the official national statistics [17]. We only used data with ACU and AMR. It has been shown that THIN is broadly representative at the national level in terms of demographics, deprivation and chronic diseases [18].

### 2.2 Coding System in Primary Care

In primary care data, MH conditions can be identified using the Read Code System, a hierarchical classification of symptoms and diagnoses [19]. These codes map to a newer coding system, SNOMED, used in UK primary care [20]. Each alphanumeric code (i.e., a unique combination of letters, numbers and dots) represents a specific symptom or diagnosis, including ICD‐10 codes. Read codes are arranged in a hierarchy, considering chapters and categories. We provide an example of how this hierarchy works in Appendix S1A. Read codes were designed to facilitate recording in clinical practice, so health personnel can recognise a code in the computer and click on it for saving in the patient history. In that way, retrospective cohorts can be constructed from which prevalence and incidence of different health conditions can be calculated. Since their creation, Read codes have been regularly updated; for this study, we used the version supplied by the THIN provider in 2019 [21].

### 2.3 Validation Process

**Figure 1:**
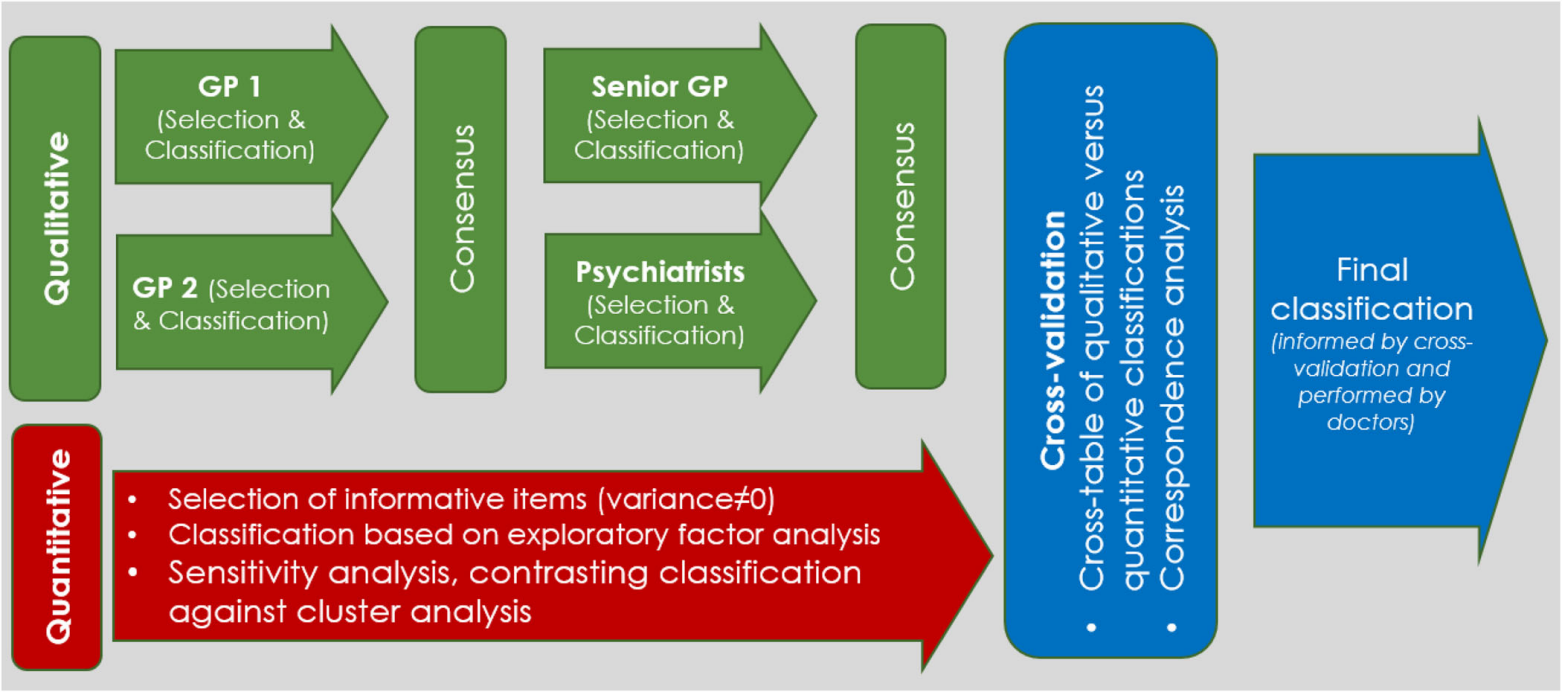
Graphical description of the validation process.

Using the higher levels of the Read codes hierarchy, we started with a comprehensive pre‐selection of Read codes relevant to MH conditions. We restricted these codes to those recorded in THIN at least once in people aged 18–99 years during the observation period (2008–2017), to remove redundant/obsolete codes. The same preselected Read codelists were provided to the six GPs and Psychiatrists for a qualitative revision (Section 2.3.1) and the statistician for performing a quantitative analysis (Section 2.3.2). The clinical revision and statistical analysis were performed independently to select the relevant Read codes and classify them into categories. Then, we contrasted results from both independent steps (cross‐validation), generating a final selection and classification informed by qualitative and quantitative criteria (Section 2.3.3). All these steps are summarised in Figure 1 and described in the following subsections.

It is important to note that these codelists were designed to be sensitive, that is, comprehensive rather than specific. They, therefore, include a wide range of codes, including diagnosis codes, symptom codes, history codes and treatment codes that infer a particular category of mental disorder. For individual studies, researchers might wish to identify a more specific subset of codes from these comprehensive lists.

#### 2.3.1 Qualitative Revision

Based on the preselected Read codes list, two GPs, D.N. and C.A., selected and classified codes into clinically relevant categories (i.e., MH conditions). Raters were asked to consider, for each code, whether it was likely to indicate the presence of one of the disorders typified in the chapter on Mental, Behavioural and Neurodevelopmental Disorders in the International Classification of Diseases and Related Health Problems (ICD). Read codes could be allocated to more than one category. This revision was independent initially, after which the GPs discussed to reach an initial consensus. Then, a Senior GP (K.W.) independently reviewed the selection and classification, resolving discrepancies and highlighting areas where coding classification decisions were less clear. Three psychiatrists (two general adults, D.O. and J.F.H., and one old age psychiatrist, C.C.) then independently reviewed the updated selection and classification. Finally, the whole panel (three GPs and three psychiatrists) resolved any remaining discrepancies or uncertainties in a final consensus step, to produce a set of codelists agreed by clinical consensus.

#### 2.3.2 Quantitative Analysis

The statistician (J.C.B.‐A., supervised by I.P.) started the analysis with the same preselected Read codes list as the doctors. First, he recovered all data related to these codes from patients aged 18–99 years who were registered in THIN between 2008 and 2017. He recorded the frequency of use of the Read codes for each patient. An exploratory factor analysis (EFA) was performed to identify clusters that allowed the classification of the selected Read codes. EFA was performed by using a Maximum Likelihood estimator, retaining all factors above an eigenvalue of 2.5. Retained factors were quickly linked to each Read code by using the factor loadings from an Oblimin rotation, generating the clusters needed for classification. The statistician performed an alternative cluster analysis to confirm the consistency of the classification generated. To ensure reproducibility, analysis details are provided in Appendix S1B.

#### 2.3.3 Quantitative and Qualitative Cross‐Validation

Independent classifications produced in the previous two steps were contrasted using correspondence analysis. A cross‐table was generated, with the qualitative classification in rows and the quantitative classification in columns. Correspondence analysis decomposes the Chi‐square statistic calculated from the cross‐table into orthogonal factors. Thus, a two‐dimension plot was generated to visualise which categories—from both the qualitative and quantitative classifications—are more or less connected. This is also useful so that quantitative categories can gain clinical meaning as long as they are graphically linked to specific qualitative categories.

Informed by the correspondence analysis results, the panel of doctors revisited their decisions about the selection and classification of Read codes. Then, the original list of MH codes from the qualitative revision (Section 2.3.1) was updated, producing a final classification of MH codes.

### 2.4 Comparing Classifications Against Psychotropic Drug Treatment

The original (Section 2.3.1) and final lists of MH codes (Section 2.3.3) were compared against appropriate psychotropic drug treatment. The assumption was that if the updated MH codelist is clinically valid, then it should recover—from the same primary care data set—more patients prescribed appropriate treatment. Drug treatment prescribed to patients was identified in THIN using the UK British National Formulary (BNF) codes, a comprehensive list of pharmaceutical drugs [22]. Doctors revised the BNF classification (based on chapters and subchapters), reaching a consensus about which MH conditions were clinically connected to which group of drugs. Then, all patients aged 18–99 years, observed from 2008 to 2017, with at least one prescription of drug treatment relevant to the specific MH condition/classification, were included. Given this population, we counted the number of patients whose records included an MH code from the original versus the final MH codelists. This analysis was performed for MH categories experiencing a noticeable change/addition of medical codes in step 2.3.3.

## 3 Results

We preselected a total of 2007 Read codes relevant to MH diseases and recorded at least once for persons aged 18–99 years during the observation period 2008–2017.

### 3.1 Qualitative Revision

From the preselected 2007 Read codes, a total of 1638 were finally selected by all doctors. They agreed on the classification of these Read codes into 12 categories of MH disorders (Table 1): severe mental illness (SMI), cognitive decline, anxiety, depression, mixed anxiety‐depression, stress‐related, deliberate self‐harm, sleeping disorders, life events, general mental health (GMH), non‐specific and other MH conditions. This initial classification slightly changed later (see point 3.3).

**Table 1:** Classification of medical codes from the qualitative revision.

Type	Number of medcodes	%	Examples of medcodes	
			Read Code	Description
Severe mental illness (SMI)	307	18.74	Eu31.00	Bipolar affective disorder
			E10.00	Schizophrenic disorders
Cognitive decline (Cog)	175	10.68	6AB.00	Dementia annual review
			Eu01.00	Vascular dementia
Anxiety (Anx)	44	2.69	E200.00	Anxiety states
			E200111	Panic attack
Depression (Dep)	150	9.16	9H92.00	Depression interim review
			E2B1.00	Chronic depression
Mixed anxiety‐depression (MAD)	5	0.31	E200300	Anxiety with depression
			Eu41200	Mixed anxiety and depressive disorder
Stress‐related (Str)	30	1.83	13HT100	Stress at home
			13JM.13	Stress at work
Deliberate self‐harm (DSH)	150	9.16	TK…15	Attempted suicide
			ZX1.13	Deliberate self‐harm
Sleeping disorders (Slp)	64	3.91	1B1B.11	Insomnia
			R005.00	Sleep disturbances
Life events (LEv)	10	0.61	13Hc.00	Bereavement
			1BE.00	Life crisis
General MH (GMH)	243	14.84	9N1T.00	Seen in psychiatry clinic
			6A6.00	Mental health review
Non‐specific (NSp)	80	4.88	1BO.00	Mood swings
			1B1J.11	Emotional upset
Other mental health conditions (Oth)	380	23.20	E21.00	Personality disorders
			E271.00	Anorexia nervosa
Total	1638	100.00		

### 3.2 Quantitative Analysis

**Figure 2:**
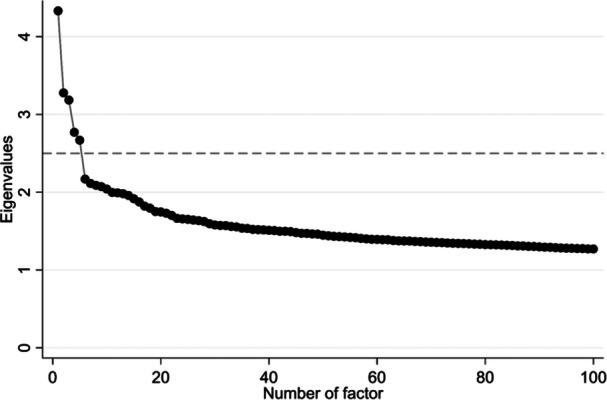
Screeplot from the exploratory factor analysis.

We produced a data set with 497,649 patients who had at least one of these Read codes (Appendix S1C). Read codes presence was coded as 1 and absence as 0, and those with < 1% of presence in the data set were removed. Thus, from the preselected 2007 Read codes, a total of 1364 were included in the EFA. In this analysis, five factors were retained from the unrotated factorial solution (Figure 2). After an Oblimin rotation, each Read code was linked to only one of the five factors, as visible in Table 2. The classification of the 1364 Read codes in five different factors was consistent with the cluster analysis performed independently, as shown in Appendix S1B.

**Table 2:** Factors retained in the exploratory factor analysis for the classification of medical codes.

Factor	Eigenvalue	Number of medical codes	Frequency of use^a^ median (interquartile range)
F1	4.33	306	426 (1799)
F2	3.28	244	599 (4043)
F3	3.18	54	1366 (5234)
F4	2.77	736	1454 (8460)
F5	2.67	24	723 (1447)

*Note:* A total of 1364 medical codes were analysed from a data set of 497,649 persons.

^a^ Median and interquartile ranges were calculated within each factor, taking the frequency of use per each medical code for 10 years.

### 3.3 Quantitative and Qualitative Cross‐Validation

**Figure 3:**
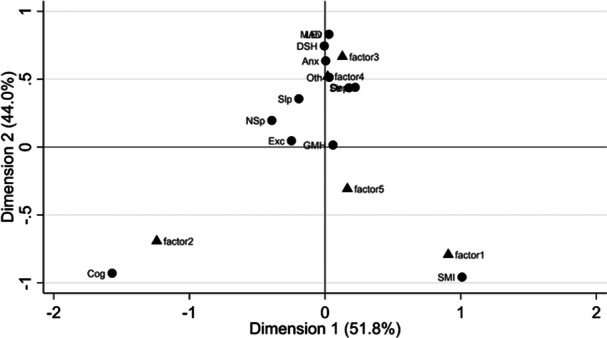
Quantitative and qualitative classification correspondence plot. Exc, excluded from the qualitative but not from the quantitative analysis. Factors 1–5 correspond to factors from the quantitative analysis (Table 2). Mental health conditions correspond to categories from the qualitative analysis and are Anxiety (Anx), Deliberate self‐harm (DSH), Cognitive decline (Cog), Depression (Dep), General MH (GMH), Sleeping disorders (Slp), Life events (LEv), Mixed Anxiety‐Depression (MAD), Non‐specific (NSp), Other Mental Health Condition (Oth), Severe Mental Illness (SMI), Stress‐related (Str).

Figure 3 shows the two‐dimension plot from correspondence analysis performed on a cross table with qualitative and quantitative classifications (Appendix S1D). The figure shows that Factor‐1 is linked to SMI while Factor‐2 is linked to cognitive decline, providing a clinical meaning to these factors statistically detected from data. For other factors, the correspondence with other clinical classifications is less clear. The quantitative selection and classification recovered several Read codes for SMI and cognitive decline that were discarded by the clinicians during the qualitative process or classified as GMH mainly non‐condition specific codes (e.g., ‘Seen by psychiatrist’, ‘Under care of mental health team’, ‘Crisis intervention’). Thus, doctors could revisit, inform and improve their original selection and classification, updating their original decisions. In brief, they recovered two medical codes previously excluded in the selection process and reclassified 350 medical codes informed by the cross‐validation results. Most of these medical codes were originally classified as GMH (244), turning into Other (75), SMI (47), cognitive decline (19), and the new categories of perinatal MH problems (17) and mental capacity (8), or just removed (85, including administrative codes). Regarding the original 12 categories (from point 3.1), mixed anxiety‐depression codes were recategorized as Depression; thus, mixed anxiety‐depression disappeared as a category together with GMH. However, the number of categories was still 12 since the new (1) perinatal MH problems and (2) mental capacity categories were added. A list with diagnosis perinatal and mental capacity medical codes, and examples of the medical codes removed is in Appendix S1E. The final classification looks as follows:

1. SMI
2. cognitive decline (which includes dementia and non‐specific codes)
3. anxiety
4. depression
5. non‐specific stress‐related
6. deliberate self‐harm and suicide
7. sleep disorders
8. life events and adjustment reactions
9. mental capacity
10. perinatal mental health problems
11. other mental health conditions (e.g., psychogenic or neurotic disorders, phobia, adjustment disorder, attention deficit hyperactivity disorder, eating disorders)
12. non‐specific mental health symptoms (e.g., emotional behavioural problems, irritability and anger, poor self‐esteem, restlessness and agitation, etc.).

Each category could include indictive diagnostic, symptom, treatment or personal history codes. A full list with the Read codes containing labels of the classification is visible in https://zenodo.org/records/10535386.

### 3.4 Comparing Classifications Against Drug Treatment

Based on the BNF classification, we generated the list of drug treatments clinically linked to the MH conditions, for example, mood stabilisers, first‐ and second‐generation antipsychotics for SMI, and antidementia drugs (Acetylcholinesterase inhibitors and Memantine) for cognitive decline. We selected a cohort of 58,490 persons with at least one prescription of antipsychotic treatment for comparison against SMI codelists and another cohort of 37,478 persons with at least one prescription of dementia treatment for comparison against cognitive decline codelists (Appendix S1C). Table 3 shows that, before the cross‐validation, the original SMI codelist matched 7689 persons prescribed antipsychotics, while the final SMI codelist—informed by the cross‐validation—matched 19,826 persons prescribed antipsychotics. The same table also shows that many patients with records originally classified as GMH were also prescribed antipsychotic or mood stabiliser prescriptions. Table 4 shows that the original cognitive decline codelist matched 30,957 persons prescribed anti‐dementia drugs, while the final cognitive decline codelist matched 31,134 persons prescribed anti‐dementia drugs. Some of those codes previously classified as GMH that turned out to be associated with original cognitive decline codes were specifically related to mental capacity. During the iterative validation process (2.3), a new category called Mental Capacity was generated to include these codes (e.g., ‘Lacks capacity to give consent (Mental Capacity Act 2005)’ and ‘Assessment of mental capacity in accord Mental Capacity Act 2005’). The comparison in Table 4 demonstrated an association of the Mental Capacity codes with prescriptions of drugs for dementia.

**Table 3:** Comparing medical codes classifications before and after the cross‐validation step in people given at least one prescription of antipsychotic treatment in 10 years of observation.

Validation step	Classification	Number of patients with one or more prescriptions			
		1st generation	Mood stabiliser	2nd generation	Total
Qualitative only (before cross‐validation)	SMI	1486	695	5508	7689
	GMH^a^	4069	2105	10,933	17,107
Qualitative informed by the cross‐validation results	SMI	4812	2261	12,753	19,826

*Note:* There were a total of 58,490 persons with at least one prescription of antipsychotic treatment in 10 years of observation.

Abbreviations: GMH, general mental health; SMI, several mental illness.

^a^ After the cross‐validation, GMH medical codes (quantitatively associated with cognitive decline Read codes) were revisited by doctors, and some of them were reclassified as SMI (last row, at the bottom).

**Table 4:** Comparing medical codes classifications before and after the cross‐validation step in people given at least one prescription of dementia treatment in 10 years of observation.

Validation step	Classification	Number of patients with one or more prescriptions		
		Acetylcholinesterase inhibitor	Glutamate antagonist	Total
Qualitative only (before cross‐validation)	Cog	23,751	7206	30,957
	NSp^a^	1156	401	1557
	Oth^a^	221	95	316
	GMH^b^	611	198	809
Qualitative informed by the cross‐validation results	Cog	23,836	7298	31,134
	Cap	611	198	809

*Note:* There were a total of 37,478 persons with at least one prescription of dementia treatment in 10 years of observation.

Abbreviations: Cap, mental capacity; Cog, cognitive decline; GMH, general mental health; NSp, non‐specific; Oth, other mental health condition.

^a^ After the cross‐validation, these medical codes (quantitatively associated with Cog Read codes) were revisited by doctors and some of them were reclassified as Cog (second row from the bottom).

^b^ After the cross‐validation, GMH medical codes (associated with Cog Read codes) were revisited by doctors and reclassified as Cap (last row, at the bottom).

## 4 Discussion

We produced comprehensive medical codelists for 12 groups of MH problems, validated using a combined qualitative and quantitative approach. The use of an iterative stepwise cross‐validation approach added value to this work and led to clinically meaningful implications. For example, it changed how we attributed some more general MH codes that could be a priori applicable to a variety of MH diagnoses, into more specific categories of MH diagnoses. This allowed for increased sensitivity of the codelists for detecting MH diagnoses that were missing from electronic primary care records, though with a probable corresponding loss of specificity.

For validating MH codelists, applying a solely qualitative or quantitative approach may not be enough to obtain sensitive or valid case definitions. The sole use of qualitative validation by consensus is the most typical in literature [13], and it is commonly based on the expertise of a small group of doctors. Although essential, their opinion cannot integrate all the preferences of primary care doctors for which codes are used in daily practice. Without integrating this information, codelists can be valid but may ignore relevant cases at the same time (lack of sensitivity). This is critical if the case definition is based on codelists exclusively. As our study showed, quantitative analysis using primary care data can summarise how doctors use available codes to inform and improve qualitative validation. Conversely, quantitative analysis alone is not sufficient for validation: it always needs the substantive guidance of clinical professionals. The cross‐validation step (Section 3.3) presented a new alternative to combine both types of validations in a practical way, which can be extended to validate other codelists. For example, a similar mixed approach can be applied to explore disorders other than SMI or dementia by granulating these categories in subtypes and mapping them with new factors/clusters detected with quantitative tools (see more comments in Appendix S1B).

The proposed mixed qualitative and quantitative validation approach avoids the need for external data, maximising benefits from internal data. When this Internal validation was performed, our final classification of MH codes was demonstrated to be consistent with the type of psychotropic drug treatment people were prescribed. This is important when the codelist is used alone or combined with drug treatment. If the codelist is used alone, having more codes to detect valid cases is valuable in studies with short observation windows because, in primary care, diagnosis or symptom codes can be underused and are less frequently recorded than drug treatments. If the codelist is used in combination with drug treatment records, then relevant cases can be recovered as well. For any of these case definitions, the mixed approach takes advantage of the rich data available to produce more sensitive case definitions, and this is done in a systematic and reproducible way.

We have identified some strengths and limitations. As far as we are aware, this is the first time that routinely collected primary care data have been used in the validation process of MH codes lists, beyond the simple analysis of frequencies of codes used by GPs. This is the first time a mixed qualitative and quantitative approach has been applied to validate lists of MH medical codes [13]. A subjective element to classification remains; some codes are challenging to classify or could belong to more than one category. We could not completely overcome the problem of undercoding; for example, some people who were prescribed a psychotropic drug had no symptom, diagnosis, administrative or other treatment medical code in their record to indicate the underlying reason for prescribing.

This study has different implications for MH research. We offer a pragmatic alternative route for future validations that do not require access to additional source data (e.g., clinic letters) or other external sources. Details of all the Read codes selection and classification—including a qualitative extra‐classification by symptoms and diagnoses are available for future users at https://zenodo.org/records/10535386. The validated codelists are a starting point for future researchers and should be refined according to the study question; for example, some might need to be merged or made more specific, if specificity is more critical than sensitivity for the study question.

In conclusion, we produced a comprehensive set of medical Read codelists for 12 MH conditions validated by a combination of a clinical consensus panel and quantitative cluster analysis with cross‐validation. The codelists are a starting point for future researchers and should be refined according to the specific study question. The mixed qualitative and quantitative approach maximises benefits from internal data, avoiding extra costs and logistics from external data collection/recovery in validating MH codelists.

## Author Contributions

Juan Carlos Bazo‐Alvarez, Kate Walters and Irene Petersen conceived the idea. Kate Walters, Christina Avgerinou, Danielle Nimmons, Joseph F. Hayes, Claudia Cooper and David Osborn performed the qualitative revision, and the final validation step informed by the quantitative revision. Juan Carlos Bazo‐Alvarez performed the quantitative revision and other statistical analyses, supervised by Irene Petersen. Juan Carlos Bazo‐Alvarez drafted the manuscript, and all authors contributed significatively to improve it up to its final version.

## Ethics Statement

THIN data, also known as IQVIA Medical Research Data, have a REC Reference 18/LO/0441 as visible on the NHS Health Research Authority website. Scientific approval to undertake this study was received from the South East Medical Research Scientific Review Committee at IQVIA (SRC Reference Number: 20SRC057). The IQVIA SRC did not request extra participants' consent for this study, and IQVIA counts with all permissions requested by the NHS Health Research Authority (including waiver of consent). All research methods were carried out in accordance with the NHS Health Research Authority guidelines and regulations.

## Conflicts of Interest

Joseph F. Hayes has received consultancy fees from the Wellcome Trust and Juli Health. The other authors declare no conflicts of interest.

## Supporting information

Supporting information.

## Data Availability

Data were analysed under THIN licence and are not available for sharing. All the validated codelists are fully available at https://zenodo.org/records/10535386.
