# Supplementary material for: Defining Mental Health Conditions Within Primary Care Data: A Validation Study With a Mixed Qualitative and Quantitative Approach
Source: J Eval Clin Pract. 2024 Dec 11;31(1):e14256. doi: 10.1111/jep.14256 (PMC11632910; doi:10.1111/jep.14256)
Supplement: Supplementary file 1 — Supporting information. [file JEP-31-0-s001.docx]

Supplemental Material

Appendix A: Read codes hierarchy example

A Infectious and parasitic diseases 0 Occupations

B Neoplasms 1 History & symptoms

C Endocrine, nutrition, metabolic and immunity disorders 2 Examination and signs

D Diseases of blood and blood forming organs 3 Diagnostic procedures

E Mental disorders 4 Laboratory procedures

F Nervous system and sense organ diseases 5 Radiology & physics in medicine

G Circulatory system diseases 6 Preventative procedures

H Respiratory system diseases 7 Operations, procedures & sites

J Digestive system diseases 8 Other therapeutic procedures

K Genitourinary system diseases 9 Administration

L Complications of pregnancy, childbirth and the puerperium

M Skin & subcutaneous tissue diseases

N Musculoskeletal and connective tissue diseases

P Congenital anomalies

Q Perinatal conditions

R Symptoms, signs and ill-defined conditions

S Injury & poisoning

T Causes of injury and poisoning

U External causes of morbidity and mortality

Z Unspecified conditions


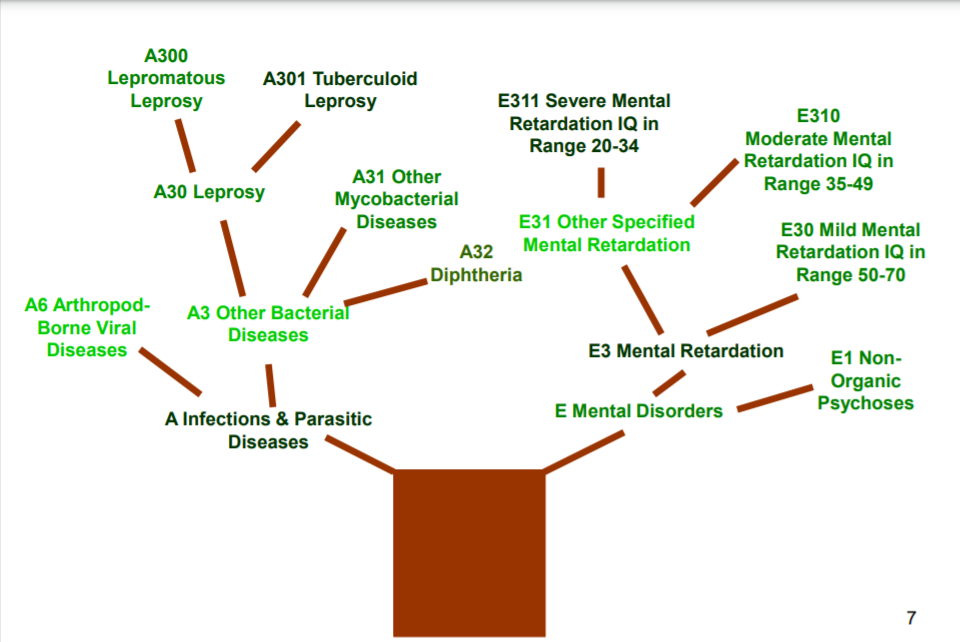


Appendix B: Details of the quantitative analyses in Section [2.3.2](#MEP_L_sec2_3_2)

We started with a dataset caontaning binary data that that represented the presence/abscense (1/0) of each of the 1,364 Read codes (in columns). Since most codes are rarely used, the analysis was particularly chanlleging (too many 0 values).

We performed the exploratory factor analysis (EFA) to identify clusters of Read codes that allowed their classification. In other words, by detecting latent factors and rotate the loadings matrix, we were able to detect which Read codes were more linked to a common factor (e.g., a specific health problem). The EFA was performed by using a Maximum Likelihood estimator, to facilitate the analysis of a dataset with a big amount of zero (0) values in a reasonable computation time. Retained factors (eigenvalue▒>▒2.5; 5 factors retained) were linked to each Read code by using the factor loadings from an Oblimin rotation, generating the clusters needed for classification.

In parallel, we performed a hierarchical cluster analysis to confirm the consistency of the classification generated by EFA. The dataset was transposed (i.e., Read codes as rows), and a distances matrix was computed where each distance was a measure of the Read codes’ proximity, i.e., Jaccard distance. The hierarchical clustering was performed by using a Euclidean linkage method. We used the Calinski/Harabasz pseudo F to define the optinal number of clusters, finding 5-6 clusters as optimal. Dendrograms were generated at different similarity levels for visual inspection. After a careful review, we found that most Read codes were grouped by hierarchical cluster in a similar way as EFA did (▒>▒80% of consistency).

We are aware there are alternative methods that could be slightly better for this data scenario; for example, EFA estimators alternative to ML or other clustering tool such as self-organising maps (SOM) or partition around medoids (PAM). We tried these and other options but they resulted ineffective to handle this type scattered data. In the end, simpler tools such as EFA-ML and hierarchical clustering provided a practical solution that covered our needs. However, in future studies focused on the validation of medical codes for a more narrowed mental health spectrum, these alternative analysis tools are still a viable option.

All analyses were performed with Stata v 17.0 and R 4.2.1.

Appendix C: Flowcharts


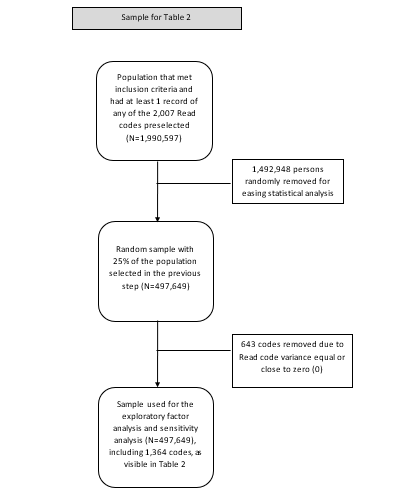


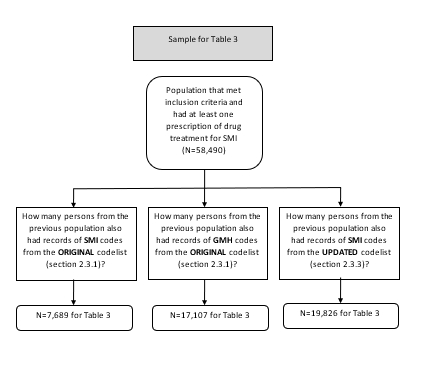


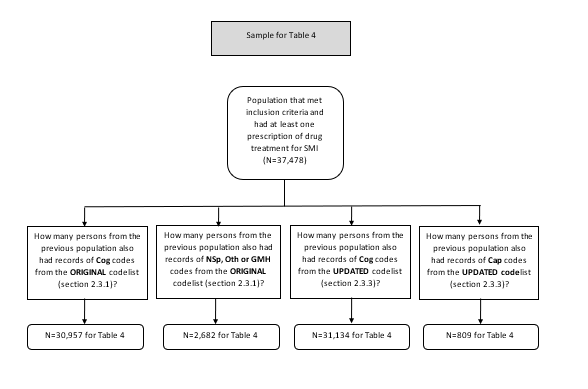


Appendix D: Cross-table for the quantitative and qualitative classifications

| Table E. Cross-table for quantitative and qualitative classification of mental health Read codes | | | | | | |
| --- | --- | --- | --- | --- | --- | --- |
| Classification agreed by doctors | Classification from exploratory factor analysis | | | | | Total |
|  | factor 1 | factor 2 | factor 3 | factor 4 | factor 5 |  |
| Anxiety (Anx) | 2 | 2 | 1 | 35 | 0 | 40 |
| **Cognitive decline (Cog)** | 3 | 130 | 1 | 12 | 2 | 148 |
| Deliberate self-harm (DSH) | 1 | 3 | 6 | 80 | 1 | 91 |
| Depression (Dep) | 24 | 4 | 21 | 92 | 2 | 143 |
| General MH (GMH) | 43 | 28 | 3 | 104 | 3 | 181 |
| Life events (LEv) | 0 | 0 | 0 | 10 | 0 | 10 |
| Mixed Anxiety-Depression (MAD) | 0 | 0 | 0 | 5 | 0 | 5 |
| Non-specific (NSp) | 3 | 18 | 0 | 46 | 2 | 69 |
| Other Mental Health Condition (Oth) | 25 | 19 | 17 | 214 | 2 | 277 |
| **Severe Mental Illness (SMI)** | 187 | 9 | 1 | 30 | 7 | 234 |
| Sleeping disorders (Slp) | 3 | 9 | 1 | 40 | 2 | 55 |
| Stress-related (Str) | 4 | 1 | 2 | 18 | 0 | 25 |
| Excluded | 11 | 21 | 1 | 50 | 3 | 86 |
| Total | 306 | 244 | 54 | 736 | 24 | 1364 |
|  | | | | | | |

Appendix E: List of diagnosis perinatal (DPe), mental capacity (Cap) and examples of removed medical codes.

| Table F. List of diagnosis perinatal (DPe), mental capacity (Cap) and examples of removed medical codes. | |
| --- | --- |
| **Diagnosis Perinatal (Dpe)** | |
| Eu53.00 | [X]Mental and behav disorders assoc with the puerperium NEC |
| L184400 | Mental disorder in puerperium - baby previously delivered |
| L184200 | Mental disorder in the puerperium - baby delivered |
| Eu53z00 | [X]Puerperal mental disorder, unspecified |
| Eu53000 | [X]Mild mental/behav disorder assoc with the puerperium NEC |
| Eu53100 | [X]Severe mental and behav disorder assoc wth puerperium NEC |
| L184300 | Mental disorder during pregnancy - baby not yet delivered |
| L184.00 | Mental disorders in pregnancy, childbirth and the puerperium |
| E204.11 | Postnatal depression |
| E03y300 | Unspecified puerperal psychosis |
| Eu53111 | [X]Puerperal psychosis NOS |
| E204.11 | Postnatal depression |
| 62T1.00 | Puerperal depression |
| 6G00.00 | Postnatal depression counselling |
| Eu53011 | [X]Postnatal depression NOS |
| Eu53012 | [X]Postpartum depression NOS |
| Eu32B00 | [X]Antenatal depression |
| **Capacity (Cap)** | |
| 28▒N..00 | Assess of mental capacity in accord Mental Capacity Act 2005 |
| 9NdL.00 | Lacks capacity to give consent (Mental Capacity Act 2005) |
| 13Im.00 | Subj cert auth issued Adults with Incapacity (Scot) Act 2000 |
| 13Io.00 | Has guardian appointd Adults with Incapacity (Scot) Act 2000 |
| 2JR..00 | Lack mental capacity make decision Mental Capacity Act 2005 |
| 9DM..00 | Incapacity (Scotland) Act 2000 certificate |
| 9DM0.00 | Incapacity (Scotland) Act 2000 certificate issued |
| 9Ng6.00 | Independent mental capacity advocate instructed |
| **Examples of removed medical codes** | |
| 1B1g.00 | No hallucinations |
| 384..00 | Psychological analysis |
| 384..11 | Psychoanalysis |
| 8HHK.00 | Referral to bereavement counsellor |
|  | |
